# Evaluation of serum pro/anti-angiogenic biomarkers in hyperglycemic rats treated with *Securigera securidaca* seeds, alone and in combination with Glibenclamide

**DOI:** 10.34172/jcvtr.32960

**Published:** 2024-03-13

**Authors:** Elham Bahreini, Mohammad Babaei, Forogh Mohammadi, Shahin Alizadeh-Fanalou

**Affiliations:** ^1^Department of Biochemistry, Faculty of Medicine, Iran University of Medical Sciences, Tehran, Iran; ^2^Department of Clinical Sciences, Faculty of Veterinary Sciences, Bu-Ali Sina University, Hamedan, Iran; ^3^Department of Veterinary, Agriculture Faculty, Kermanshah Branch, Islamic Azad University, Kermanshah, Iran; ^4^Department of Clinical Biochemistry, School of Medicine, Urmia University of Medical Sciences, Urmia, Iran

**Keywords:** Angiogenesis, Diabetes, Glibenclamide, *Securigera Securidaca*, VEGF

## Abstract

**Introduction:**

Herbal medicines are commonly used by many people with diabetes in addition to standard treatment. Plants contain numerous known and unknown compounds that may exacerbate or ameliorate diabetes complications. Therefore, it is crucial to be aware of the side effects of these herbs before prescribing them. This study aimed to investigate the effects of hydroalcoholic extracts of *Securigera securidaca* (HESS) seeds alone and in combination with glibenclamide on the angiogenic/anti-angiogenic balance in streptozotocin (STZ)-induced diabetic rats.

**Methods:**

Groups involved in this animal study included diabetic and healthy controls, three doses of HESS, glibenclamide, and combination therapy. Serum samples were collected and analyzed for a vascular endothelial growth factor (VEGF), fibroblast growth factor 21 (FGF21), fetal liver kinase 1 (FLK-1), soluble fms-like tyrosine kinase 1 (sFLT-1), and transforming growth factor -beta (TGF-β).

**Results:**

Induction of diabetes increased VEGF, FGF21, and TGF-β serum levels and decreased circulating FLK-1 and sFLT-1 factors. Herbal extract, except TGF-β, had little effect on the above blood levels even at the highest doses. Glibenclamide was more effective than the highest dose of HESS in improving the vascular complications of diabetes. Combination therapy with the highest dose of HESS partly enhanced the glibenclamide effects.

**Conclusion:**

Compared with glibenclamide as a standard chemical drug, HESS had no significant effects on the blood levels of the pro/anti-angiogenesis factor in diabetic rats. Glibenclamide attenuated the levels of the biomarkers and its effects were somewhat enhanced in combination with the highest dose of HESS.

## Introduction

 Vascular disorders are the leading cause of myocardial infarction, kidney failure, blindness, and poor wound healing in diabetes. Such complications in diabetes are often due to progressive disorders of small, medium, and large arteries, which increase the risk of peripheral ischemia. Under such conditions, the normal tissue response is to form new blood vessels from larger arteries to minimize ischemic damage, conditions that are mainly observed in the myocardium and retina of diabetics.^1^ Under normal conditions, angiogenesis is controlled by angiogenic and antiangiogenic stimuli, but in diabetes, the balance between these factors may be disturbed. It appears that treatment with antidiabetic drugs affects this balance.

 It is well-studied that the upregulation of VEGF in diabetes leads to abnormal angiogenesis and tissue damage as seen in nephropathy and retinopathy. Despite this phenomenon, a decrease in VEGF expression has been reported in diabetic cardiomyopathy, which is associated with a decrease in capillary density, an increase in fibrosis, and a decrease in cardiac contractility. Also, a decrease in the expression of VEGF has been reported in experimental studies on skin wounds created in diabetic animal models.^2^ The important effect of VEGF in vascular regeneration is mediated by binding to its receptors, fms-like tyrosine kinase 1 (FLT-1) and fetal liver kinase 1 (FLK-1), and is inhibited by the naturally occurring circulating form of FLT-1 or Soluble fms-like tyrosine kinase 1 (sFLT-1). sFLT-1 or VEGF receptor-1 is a dissociated fragment of the FLT-1 receptor that acts as a VEGF antagonist and potential inhibitor of angiogenesis. Therefore, changes in plasma levels of VEGF and sFLT-1 may be related to the severity of vascular complications of diabetes. It has been shown that the FLT-1 receptor is essential for the interaction between endothelial cells and cell matrix, while the FLK-1 receptor regulates the differentiation and mitogenesis of endothelial cells.^3^

 Plant phenolic or polyphenolic compounds as natural secondary metabolites have attracted the attention of researchers due to their medicinal properties, including antioxidant, anti-inflammatory, antimicrobial, pro/anti-angiogenic, antidiabetic, cardioprotective, liver protective, and neuroprotective activities.^4,5^

 Polyphenolics, which are divided into flavonoids and non-flavonoids, are benzene derivatives with carboxylic, hydroxyl and/or methoxyl groups.^6^ Several studies have demonstrated both anti- and proangiogenic properties of such phytochemicals with the possible effects on involved factors such as VEGF, HIF-1α, FGF21, TGF, and inflammatory agents.^6-8^

*Securigera securidaca* (L.) Degen & Dorfl (*S. securidaca*),with local names of *Adasol-molk* and *Bitter-lentils,* belongs to the *Fabaceae *family. The seed of the plant has been used in Iranian folk medicine and also by Egyptians and Indians since ancient times to treat several ailments such as diabetes, hyperlipidemia, and hypertension.^9-11^ In a chain study, we examine the positive or negative side effects of *S. securidaca* on diabetes complications. Our previous experiment showed that the herbal extract as a supplement could improve the hypoglycemic, antioxidant, and anti-inflammatory properties of the standard drug.^11^ In this research, the possible effect of *S. securidaca* seeds on neovascularization was evaluated in the diabetic animal model, alone and in combination with glibenclamide.

## Materials and Methods

###  Seed extract preparation and determination of total phenolic and flavonoid content 

 As mentioned above, the present study is a continuation of our previous studies on the side effects of *S. securidaca* seeds with the aim of investigating its effect on the phenomenon of angiogenesis in diabetes.* S. securidaca* seed (herbarium code of PMP-756) extraction and determination of total phenolic and flavonoid contents have been described in detail in our previous publication.^11^ Briefly, the ground seed was extracted with 70% ethanol and concentrated by rotary evaporation. The total phenolic content of the hydroalcoholic extract was determined using the Folin Ciocalteau reagent according to the method of Singleton and Rossi.^12^ The total flavonoid content of the crude extract was estimated using the aluminum chloride colorimetric method and quercetin as a standard.^13^ GC-MS and HPLC analysis of hydroalcoholic extracts of S. securidaca seeds by Aldal’in et al showed high contents of flavonoids, tannins, saponins, and alkaloids. In addition, the phytochemical analysis revealed high levels of L-ascorbic acid, aromatic and dodecanedioic acid derivatives, as well as β-sitosterol and oxygenated hydrocarbons.^14^

###  Experimental animals 

 40 male Wistar rats (233-247g) with normal blood sugar (90.6 ± 5 mg/dl).^15^ were obtained from the experimental study center of Iran University of Medical Sciences. After isolating 5 rats as healthy controls, diabetes was induced in the remaining rats by intraperitoneal injection of streptozotocin (STZ) (55 mg/kg-BW). After 72 houres, the blood sugar levels of STZ injected animals were assayed by a glucometer (*Emperor,* OK2-AJ). Rats with blood sugar above 250 mg/dL were considered as diabetic model. The ethics committee of Iran University of Medical Sciences confirmed that the present study and animal managment were in accordance with the guidelines of the laboratory animal care department of Iran University of Medical Sciences (Ethical code: IR.IUMS.REC 1396.31864).

###  Experimental design

 Three doses of of hydroalcoholic extracts of *Securigera securidaca* (HESS), 100, 200, and 400 mg/kg-BW.^10^ and one dose of glibenclamide (Chemidarou 5 mg/kg-BW tab, Iran).^16^ were considered for administration. The experimental groups (5 rats/group, n = 5) were: group I as Normal Control (NC) or Healthe Control (HC), group II as Diabetic Control (DC), groups III to V as diabetic rats treated with HESS doses of 100 (E-100), 200 (E-200) and 400 (E-400) mg/kg-BW, respectively, group VI treated with glibenclamide (G) 5 mg/kg-BW and groups VII and VIII treated with both G and HESS (200 and 400 mg/kg-BW) as G + HESS-200 and G + HESS-400 groups, respectively. The treatment period was 35 days and the groups were gavaged daily by 500 µl of the respective drug dose. At the end of the study, the animals were placed in a closed chamber with semi-saturated chloroform one by one, anaesthetized and then the blood samples were collected by cardiac puncture. Then, blood samples were centrifuged at 3000 rpm for 10 minutes. the sera were separated and stored at -20°C until use.

###  Biochemical analysis

 Serum Insulin (MBS724709), VEGF (MBS843480), TGF-β (MBS260302), FGF21 (MBS2024083), sFLT-1 (MBS2602003), and FLK (MBS282637) were assayed using Rat ELISA Kits from MyBioSource Company according to the manufacturer’s instructions and using a multi-plate ELISA reader (ELISA Reader-DANA-320., Japan).

 Detection of total NO was performed spectrophotometrically using rat nitric oxide assay Kit (ZellBio GmbH, Germany, a lot: ZB-NO-48A). Due to the extremely short physiological half-life of this gaseous free radical, NO was quantified on the basis of its metabolites nitrite and nitrate using the Griess method (with some modifications).^17,18^

###  Statistical analysis

 The normality of the data was verified by Shapiro-Wilk using SPSS software, version 25 (IBM Corp., Armonk, N.Y., USA). Then one way-ANOVA followed by Tukey’s test was used to determine differences between individual groups. The data were expressed as means ± standard deviation (SD). Association between parameters was evaluated by Simple Linear Regression. The significance level was set at *P* < 0.05.

## Results

**Figure 1:**
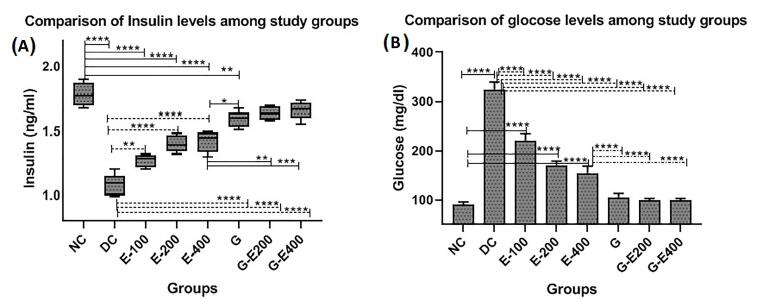
Comparison of Insulin (A) and glucose (B) levels among experimental and control groups. HESS (hydroalcoholic extract of *S. securidaca* seeds); G (glibenclamide); DC (diabetic control); NC (normal control, n = 5.( The experimental groups were 5 rats/group (n = 5); Values are means ± SD. * Significant difference degree among groups

Figure 1 shows the antiparallel changes in blood insulin compared to the changes in blood glucose levels in the study groups (ANOVA: *P* < 0.001). HESS significantly and dose-dependently increased insulin levels and decreased serum glucose concentrations in diabetic rats (*P* < 0.05). Glibenclamide was more effective than the highest dose of HESS in this regard (*P* < 0.05), and its combination with HESS partly enhanced its effect on increasing serum insulin and decreasing serum glucose levels of diabetic rats (*P* > 0.05).

###  Effects of treatments on angiogenesis/anti-angiogenesis Biomarkers

**Figure 2:**
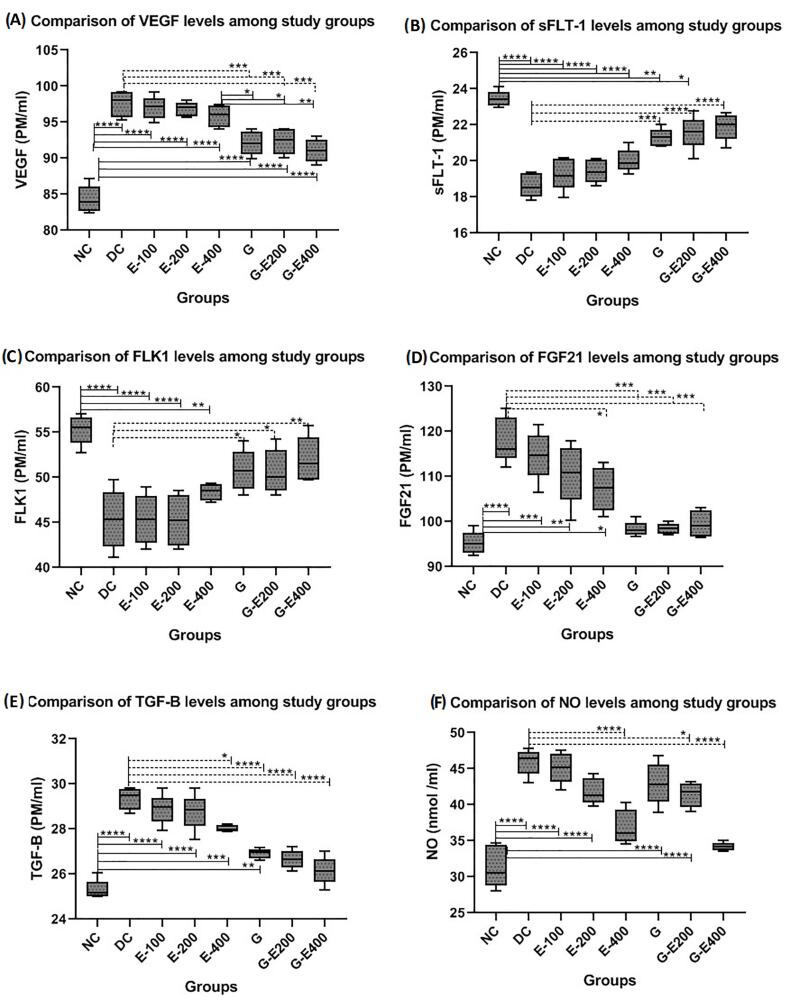
comparison of pro/ anti-angiogenic factors levels among experimental and control groups. (A) VEGF (Vascular endothelial growth factor), (B) sFLT-1 (soluble fms-like tyrosine kinase), (C) FLK-1 (fetal liver kinase 1), (D) FGF21(fibroblast growth factor 21), (E) TGF-β (transforming growth factor-beta), (F) NO (nitric oxide). HESS (hydroalcoholic extract of *S. securidaca* seeds); G (glibenclamide); DC (diabetic control); NC (normal control, n = 5.( The experimental groups were 5 rats/group (n = 5); Values are means ± SD. * Significant difference degree among groups

Figure 2 shows changes in serum levels of angiogenic/ anti-angiogenic biomarkers in control and treated groups. One-way ANOVA revealed significant differences in the serum levels of VEGF, FLK-1, sFLT-1, FGF21, and TGF-β (*P* < 0.01) between study groups. In the DC group, the serum concentrations of VEGF and FGF21, and TGF-β were significantly increased (*P* < 0.01), while serum levels of FLK-1 and sFLT-1 were significantly decreased (*P* < 0.01) compared to healthy controls. The results showed that treatment with different doses of HESS slightly altered serum levels of VEGF, sFLT-1, FLK-1 and TGF-β in a dose-dependent manner in diabetic rats, but only reduced TGF-β levels in treatment with the highest dose of HESS (400mg/kg-BW) became statistically significant compared to DC and two lower doses of HESS (*P* < 0.01). Administration of HESS resulted in a dose-dependent reduction in circulating levels of FGF21, but this reduction was not statistically significant compared to the DC group (*P* > 0.05). Compared to the highest dose of HESS, glibenclamide significantly decreased the serum levels of VEGF and TGF-β (*P* < 0.01), and non-significantly increased the serum levels of FGF-21, FLK-1 and sFLT-1 (*P* > 0.05) in diabetic rats; however, except with FGF-21, the levels of the mentioned parameters were still differ from those in the healthy group (*P* < 0.05).

 According to the data, it seems that combination therapy with glibenclamide and the highest dose of HESS partially enhanced the effects of glibenclamide on reducing the concentrations of angiogenic factors (VEGF, FGF21, and TGF-β) and increasing the levels of antiangiogenic agents (FLK-1 and sFLT-1), but these improved effects were not statistically significant (*P* > 0.05) except with TGF-β (*P* = 0.02). However, combination therapy altered the concentrations of FGF21, sFLT1 and FLK-1 to a comparable extent to the NC group (*P* > 0.05). NO levels significantly increased in the DC group compared to the NC group (*P* < 0.05). Administration of the highest dose of HESS could effectively reduce NO production in diabetic rats (*P* < 0.05) and concomitant use with glibenclamide further reduced NO levels to a comparable extent in the NC group (*P* > 0.05). The effect of glibenclamide alone on the NO levels was comparable to the middle dose of HESS (200mg/kg-BW) (*P* > 0.05).

###  Correlations between study biomarkers

 Regression analysis (Table 1) showed the association between the study’s biomarkers via positive (+) or negative (-) correlation coefficients, which were significant at *P* < 0.05. As it is indicated in Table 1, VEGF levels were positively correlated with the parameters of TGF-β, FGF21 and NO (*P* < 0.001), and negatively correlated with FLK-1 and sFLT-1 (*P* < 0.001).

**Table 1:** Correlation matrix of the study variables

	**1**	**2**	**3**	**4**	**5**
1. VEGF					
2. TGF-β	0.897^*^				
3. FGF-21	0.760^*^	0.847^*^			
4. FLK-1	-0.864^*^	-0.880^*^	-0.694^*^		
5. sFLT-1	-0.844^*^	-0.858^*^	-0.699^*^	0.773^*^	
6. NO	0.671^*^	0.709^*^	0.639^*^	-0.717^*^	-0.658^*^

VEGF (Vascular endothelial growth factor), sFLT-1 (soluble fms-like tyrosine kinase), FLK-1 (fetal liver kinase 1), FGF21(fibroblast growth factor 21), TGF-β (transforming growth factor-beta), NO (nitric oxide). *P < 0.001.

## Discussion

*S. securidaca* is widely used in Iranian folk medicine to treat diabetics along with the mainstream treatments (herbal and standard drug combination). *S. securidaca,* like other plants, contains a well-known and unknown set of compounds that may have positive or negative side effects. In our previous study, antidiabetic and antioxidant properties of hydroalcoholic seed extract of *S. securidaca* were evaluated in STZ-induced diabetic rats, and the results were compared with the effects of the standard drug of glibenclamide, alone and in combination with HESS.^11^ We experienced that HESS reduced blood sugar and insulin resistance dose-dependently, and the effect was enhanced when used in combination with glibenclamide. Our previous study showed that HESS was more effective than glibenclamide in lowering the body oxidative state and pro-inflammatory cytokines such as hs-CRP and TNF-α, and raising antioxidant capacity.^11^ In this study, we assessed the possible effects of HESS on angiogenesis as the most critical process in the occurrence of diabetes complications and compared the results with the effects of glibenclamide.

 In agreement with the previous studies, induction of diabetes increased circulating levels of VEGF, FGF21, and TGF-β and decreased plasma concentrations of FLK-1 and sFLT-1 in STZ-treated rats.^19,20^ In addition to the effect on its secreted tissue as a paracrine factor, VEGF enters circulation and can exert its effects on other tissues that express high levels of its receptors. Therefore, lowering blood levels of VEGF could be considered as a therapeutic target. Circulating FLK-1 and sFLT-1 as natural VEGF attenuators can attache to VEGF and neutralize it.^21^ Administration of HESS did not show a protective effect in preventing an increase in serum VEGF concentration and a decrease in its soluble receptors, FLK-1 and sFLT-1 in diabetic rats. While HESS is rich in phenolic and flavonoid compounds (93.3 ± 1.5 mg GAE/g (DW) and 46 ± 1.7 mg QE /g (DW), respectively),^11^ these findings are inconsistent with the results of some studies that have stated that polyphenolic compounds downregulate the expression of angiogenic factors as an index of neovascularization.^22,23^ Park et al reported that polyphenols prevented the hypoxia-induced angiogenesis by reducing HIF-α transcriptional activity without altering its mRNA level, leading to reduce the expression of HIF-α downstream targets, including VEGF.^24^ Cerezo et al showed that VEGF-receptors were potently inhibited by some specific polyphenols.^25^ They stated the polyphenols appeared to be attached to the similar region of VEGF involved in the interaction with its receptor, and the potent inhibitory was proportional to the specificity and binding affinities. Although HESS dose of 400mg/kg.BW was a sufficient amount to lower blood sugar and prevent from oxidative stress caused by hyperglycemia, it may be a low dose in modulating the factors involved in angiogenesis. In this regard, glibenclamide was more effective than the highest dose of HESS in preventing an increase in serum VEGF levels and a decrease in circulating FLK-1 and sFLT-1 in diabetic rats. Serum levels of FLK-1 and sFLT-1 in diabetic group trated with both glibenclamide and E-400 were relatively comparable to those in healthy rats, but there was still a significant difference in VEGF values. Kimura et al in their study on sulphonylureas treatment and diabetic retinopathy, reported that glibenclamide could not downregulate VEGF and inhibit ischemia-induced retinal neovascularization.^26^ Therefore, according to our results, it can be claimed that glibenclamide may reduce blood VEGF via the upregulation of circulating FLK-1 and sFLT-1 and the combination therapy with the highest dose of HESS partly enhanced its protective effect.

 The highest dose of HESS effectively prevented the increase in NO levels in diabetic rats, while glibenclamide had little effect in this regard. Combination therapy with glibenclamide partially enhanced the effect of HESS in reducing NO levels. Recent studies have shown that NO is involved in the activation of HIF-1α through a nonhypoxic pathway that leads to VEGF upregulation.^27^ Our results also showed such a correlation between NO and VEGF levels. Such a correlation has also been reported in tumor studies, such as the report by Barbieri et al who claimed that VEGF can induce the production of eNOS and NO.^28^ However it is northworthy to say, NO shows an opposite and double-behavior in physiological condition: As a protective agent against ROS, it scavenges free radicals and as an oxidizing agent, it produces ONOO-. Increase in NO level in diabetic rats as seen in this study compared to healthy group may attribiuted to thr overproduction of ROS which reduces the bioavailability and bioactivity of NO; such a reduction in NO availability induces the expression of both iNOS and eNOS.^29^ However, the conflicting reports on blood NO concentrations in diabetes may be due to the very short physiological half-life of NO, which makes it difficult to measure.

 The highest dose of HESS and glibenclamide showed inhibitory effects on increasing serum TGF-β levels in diabetic rats, and their combined administration enhanced the effect of each on reducing serum TGF-β concentrations to the comparable extent with the levels in non-diabetic rats. Upregulation of TGF-β in long-term hypoglycemic condition plays an important role in neovascularization by stimulating the expression of angiogenic factors such as VEGF and pro-inflammatory cytokines such as TNF-α.^30^ It is a pro-fibrotic mediator that enhances the formation of extracellular matrix in fibrotic diseases like retinopathy and nephropathy.

 FGF21 is a multifunctional factor that can be produced in the liver under hyperglycemic conditions as a compensatory mechanism for maintaining metabolic homeostasis. In these conditions, it stimulates insulin secretion and reduces insulin resistance and acts as a hypoglycemic, hypolipidemic, ketogenic, and antioxidant inducer agent.^31^ Correction of hyperglycemia by injecting insulin, standard drugs, or herbal medicine can lower serum FGF21 levels.^11,32^ Lin et al in their study on the effects of FGF21 on renal fibrosis, reported that FGF21 prevents renal fibrosis via the downregulation of the TGF-β.^33^ However, no direct effect of FGF21 on VEGF expression has been reported so far. Here, despite high levels of FGF21 in diabetic control rats, elevated levels of VEGF and TGF-β may be due to several factors contributing to increased production of angiogenic factors in uncontrolled diabetes, and FGF21 alone may not be sufficient in prevention. This relationship is justified via the positive correlation of FGF21 levels with angiogenic factors in the regression results. In this regard, glibenclamide decreased TGF-β and FGF21 levels in diabetic rats more effectively than the highest dose of HESS, and combination therapy with the highest dose of HESS partially enhanced the effectiveness of glibenclamide. FGF21 exerts parts of its role in modulating energy hemostasis via the increase in circulating adiponectin, which now approved that suppresses retinal and choroidal ocular neovascularization. Adiponectin also suppresses TNF-α transcription through mRNA instability, which results in a decrease in the retinal and choroidal neovascularization.^34^

 Overall, our results showed positive correlations between angiogenesis-promoting factors and antiangiogenic factors, but a negative relationship between these two groups of parameters. Diabetes was associated with increased angiogenesis through an increase in VEGF, TGF-β, FGF-21, TNF-α and NO levels and a decrease in FLK-1 and sFLT-1 levels. Compared with glibenclamide, HESS at its highest dose had a positive but weak role in preventing angiogenesis, and the combination of both somewhat increased the efficacy of the standard drug. Despite the high concentration of phenolic and flavonoid compounds in HESS, the weak antiangiogenic effect could be due to the low concentration of HESS used in this study or the presence of inhibitors in the composition of HESS. In addition, due to the anti-diabetic properties of HESS, no negative interaction between the plant extract and the standard drug was observed in the combination therapy, a method of consumption that may be used by diabetic patients. Therefore, future studies using higher doses of HESS are recommended. However, before treatment with higher doses, LD50 values, as well as the presence of interfering derivatives in the extract should be specified. The main limitations of this study was the general examination of factors related to angiogenesis, which may be inconsistent with the above results in local tissue examinations. Therefore, this study requires future tissue studies at the level of different organs, in particular the diabetic heart, kidney and retina.

## Conclusion

 Compared with glibenclamide as a standard chemical drug, HESS had no significant effects on the blood levels of the pro/anti-angiogenesis factor in diabetic rats. Glibenclamide attenuated the levels of the biomarkers and its effects were somewhat enhanced in combination with the highest dose of HESS.

## Acknowledgments

 This project was supported by Iran University of Medical Sciences with research No: 27831. We thank the members of the Biochemistry Department of Iran University of Medical Sciences for their favors.

## Competing Interests

 There are no conﬂicts of interest to declare.

## Ethical Approval

 Animal managment was approved by Ministry of Health of Iran (IR.IUMS.REC 1396.31864) and carried out in accordance with the guidelines of the laboratory animal care department of Iran University of Medical Sciences.

## Funding

 This project was funded and supported by Iran University of Medical Sciences (Grant No. 27831).

## References

[1] Cade WT (2008). Diabetes-related microvascular and macrovascular diseases in the physical therapy setting. Phys Ther.

[2] Ferrari G, Cook BD, Terushkin V, Pintucci G, Mignatti P (2009). Transforming growth factor-beta 1 (TGF-beta1) induces angiogenesis through vascular endothelial growth factor (VEGF)-mediated apoptosis. J Cell Physiol.

[3] Amano H, Kato S, Ito Y, Eshima K, Ogawa F, Takahashi R (2015). The role of vascular endothelial growth factor receptor-1 signaling in the recovery from ischemia. PLoS One.

[4] Sun Q, Heilmann J, König B (2015). Natural phenolic metabolites with anti-angiogenic properties - a review from the chemical point of view. Beilstein J Org Chem.

[5] Ataie A, Sabetkasaei M, Haghparast A, Hajizadeh Moghaddam A, Kazeminejad B (2010). Neuroprotective effects of the polyphenolic antioxidant agent, curcumin, against homocysteine-induced cognitive impairment and oxidative stress in the rat. Pharmacol Biochem Behav.

[6] Li AN, Li S, Zhang YJ, Xu XR, Chen YM, Li HB (2014). Resources and biological activities of natural polyphenols. Nutrients.

[7] Parveen A, Subedi L, Kim HW, Khan Z, Zahra Z, Farooqi MQ (2019). Phytochemicals targeting VEGF and VEGF-related multifactors as anticancer therapy. J Clin Med.

[8] He Z, Chen AY, Rojanasakul Y, Rankin GO, Chen YC (2016). Gallic acid, a phenolic compound, exerts anti-angiogenic effects via the PTEN/AKT/HIF-1α/VEGF signaling pathway in ovarian cancer cells. Oncol Rep.

[9] Garjani A, Fathiazad F, Zakheri A, Allaf Akbari N, Azarmie Y, Fakhrjoo A (2009). The effect of total extract of Securigerasecuridaca L seeds on serum lipid profiles, antioxidant status, and vascular function in hypercholesterolemic rats. J Ethnopharmacol.

[10] Rajaei Z, Hadjzadeh M, Moradi R, Ghorbani A, Saghebi A (2015). Antihyperglycemic and antihyperlipidemic effects of hydroalcoholic extract of Securigerasecuridaca seeds in streptozotocin-induced diabetic rats. Adv Biomed Res.

[11] Alizadeh-Fanalou S, Babaei M, Hosseini A, Azadi N, Nazarizadeh A, Shojaii A (2020). Effects of Securigerasecuridaca seed extract in combination with glibenclamide on antioxidant capacity, fibroblast growth factor 21 and insulin resistance in hyperglycemic rats. J Ethnopharmacol.

[12] Singleton VL, Rossi JA (1965). Colorimetry of total phenolics with phosphomolybdic-phosphotungstic acid reagents. Am J Enol Vitic.

[13] Chang CC, Yang MH, Wen HM, Chern JC (2002). Estimation of total flavonoid content in propolis by two complementary colorimetric methods. J Food Drug Anal.

[14] Aldal’in HK, Al-Mazaideh G, Al-Nadaf AH, Al-Rimawi F, Afaneh AT, Marashdeh A (2020). Phytochemical constituents of Securigerasecuridaca seed extract using GS-MS and HPLC. Trop J Nat Prod Res.

[15] Khomari F, Kiani B, Alizadeh-Fanalou S, Babaei M, Kalantari-Hesari A, Alipourfard I (2023). Effectiveness of hydroalcoholic seed extract of Securigerasecuridaca on pancreatic local renin-angiotensin system and its alternative pathway in streptozotocin-induced diabetic animal model. Oxid Med Cell Longev.

[16] Kim JD, Kang SM, Seo BI, Choi HY, Choi HS, Ku SK (2006). Anti-diabetic activity of SMK001, a poly herbal formula in streptozotocin induced diabetic rats: therapeutic study. Biol Pharm Bull.

[17] Bryan NS, Grisham MB (2007). Methods to detect nitric oxide and its metabolites in biological samples. Free Radic Biol Med.

[18] Schulz K, Kerber S, Kelm M (1999). Reevaluation of the Griess method for determining NO/NO2- in aqueous and protein-containing samples. Nitric Oxide.

[19] Gupta N, Mansoor S, Sharma A, Sapkal A, Sheth J, Falatoonzadeh P (2013). Diabetic retinopathy and VEGF. Open Ophthalmol J.

[20] Tsuchida K, Makita Z, Yamagishi S, Atsumi T, Miyoshi H, Obara S (1999). Suppression of transforming growth factor beta and vascular endothelial growth factor in diabetic nephropathy in rats by a novel advanced glycation end product inhibitor, OPB-9195. Diabetologia.

[21] Krysiak O, Bretschneider A, Zhong E, Webb J, Hopp H, Verlohren S (2005). Soluble vascular endothelial growth factor receptor-1 (sFLT-1) mediates downregulation of FLT-1 and prevents activated neutrophils from women with preeclampsia from additional migration by VEGF. Circ Res.

[22] Cerezo AB, Winterbone MS, Moyle CW, Needs PW, Kroon PA (2015). Molecular structure-function relationship of dietary polyphenols for inhibiting VEGF-induced VEGFR-2 activity. Mol Nutr Food Res.

[23] Mirossay L, Varinská L, Mojžiš J (2017). Antiangiogenic effect of flavonoids and chalcones: an update. Int J Mol Sci.

[24] Park JJ, Hwang SJ, Park JH, Lee HJ (2015). Chlorogenic acid inhibits hypoxia-induced angiogenesis via down-regulation of the HIF-1α/AKT pathway. Cell Oncol (Dordr).

[25] Cerezo AB, Hornedo-Ortega R, Álvarez-Fernández MA, Troncoso AM, García-Parrilla MC (2017). Inhibition of VEGF-induced VEGFR-2 activation and HUVEC migration by melatonin and other bioactive indolic compounds. Nutrients.

[26] Kimura T, Takagi H, Suzuma K, Kita M, Watanabe D, Yoshimura N (2007). Comparisons between the beneficial effects of different sulphonylurea treatments on ischemia-induced retinal neovascularization. Free Radic Biol Med.

[27] Kuwabara M, Kakinuma Y, Ando M, Katare RG, Yamasaki F, Doi Y (2006). Nitric oxide stimulates vascular endothelial growth factor production in cardiomyocytes involved in angiogenesis. J Physiol Sci.

[28] Barbieri A, Palma G, Rosati A, Giudice A, Falco A, Petrillo A (2012). Role of endothelial nitric oxide synthase (eNOS) in chronic stress-promoted tumour growth. J Cell Mol Med.

[29] Aly MI, Abdalla MN, El Akad MH, El-Sheikh SA, Yousief EM (2013). Role of iNOS and eNOS expression in a group of Egyptian diabetic and nondiabetic nephropathy patients. Egypt J Intern Med.

[30] Maloney JP, Gao L (2015). Proinflammatory cytokines increase vascular endothelial growth factor expression in alveolar epithelial cells. Mediators Inflamm.

[31] Jimenez V, Jambrina C, Casana E, Sacristan V, Muñoz S, Darriba S (2018). FGF21 gene therapy as treatment for obesity and insulin resistance. EMBO Mol Med.

[32] Emanuelli B, Vienberg SG, Smyth G, Cheng C, Stanford KI, Arumugam M (2014). Interplay between FGF21 and insulin action in the liver regulates metabolism. J Clin Invest.

[33] Lin S, Yu L, Ni Y, He L, Weng X, Lu X (2020). Fibroblast growth factor 21 attenuates diabetes-induced renal fibrosis by negatively regulating TGF-β-p53-Smad2/3-mediated epithelial-to-mesenchymal transition via activation of AKT. Diabetes Metab J.

[34] Fu Z, Gong Y, Liegl R, Wang Z, Liu CH, Meng SS (2017). FGF21 administration suppresses retinal and choroidal neovascularization in mice. Cell Rep.

